# Seeking evidence of multidisciplinarity in environmental geochemistry and health: an analysis of arsenic in drinking water research

**DOI:** 10.1007/s10653-017-9919-4

**Published:** 2017-02-24

**Authors:** Abiodun D. Aderibigbe, Alex G. Stewart, Andrew S. Hursthouse

**Affiliations:** 1 Institute of Biomedical and Environmental Health Research, School of Science and Sport, University of the West of Scotland, Paisley, PA1 2BE UK; 2 Cheshire and Merseyside Public Health England Centre, Liverpool, L1 1JF UK; 3 Present Address: Department of Chemistry, Federal University of Technology, Akure, P.M.B. 704 Ondo state Nigeria; 4 Present Address: College of Life and Environmental Science, University of Exeter, Exeter, UK

**Keywords:** Multidisciplinary, Research design, Arsenic, Water, Health

## Abstract

A multidisciplinary approach to research affords the opportunity of objectivity, creation of new knowledge and potentially a more generally acceptable solution to problems that informed the research in the first place. It increasingly features in national programmes supporting basic and applied research, but for over 40 years, has been the arena for many research teams in environmental geochemistry and health. This study explores the nature of multidisciplinary research in the earth and health sciences using a sample selected from co-authored articles reporting research on arsenic (As) in drinking water from 1979 to 2013. A total of 889 relevant articles were sourced using the online version of the science citation index—expanded (SCI-expanded). The articles were classified according to author affiliation and later by author discipline/research interests using the Revised Field of Science and Technology Frascati manual DSTI/EAS/STP/NESTI (2006) 19/FINAL and a decision algorithm. Few articles were published on the topic until 2000. More articles were published across all affiliations in the last 10 years of the review period (2004–2013) than in the first 10 years (1979–1988). Only 84 (~9%) articles fell within the “earth and health” only and “earth, health and other” categories when classification was undertaken by author affiliation alone. This suggests that level of collaboration between earth and health scientists in arsenic in drinking water research may be very low. By refining the classification further using author discipline/research interests, only 28 of the 84 articles appear to be co-authored by earth and health scientists alongside professionals in other fields. More than half of these 28 articles involved descriptive non-experimental, observational study designs, limited in direct causal hypotheses and mechanistic investigation. If collaborative research is to lead to the increased multidisciplinary research, early interaction should be encouraged between students from different disciplines. In order to achieve multidisciplinarity in practise, it is imperative that scientific communities and research agencies do more to encourage interaction and integration between researchers from different disciplines. This must develop from educational institutions seeing opportunities to improve graduate skills in an increasingly diverse research landscape.

## Introduction

In order to understand and solve complex environmental and human health problems in a robust manner, we need to apply skills which transcend single disciplines. This is one reason why a multidisciplinary approach is encouraged by national funding agencies and scientific communities in the environmental sciences and other research fields (GlobalHigherEd 2009; Uiterkamp and Vlek 2007; UNICEF 2013; Armienta and Segovia 2008; Khan et al. 2010). Multidisciplinarity is becoming recognized as the source of economic and social resilience by many governments worldwide.

The Society for Environmental Geochemistry and Health (SEGH) is a multidisciplinary community of professionals and students working in the broad fields of environmental geochemistry and health, human, animal and plant. From its establishment in 1971, it has encouraged multidisciplinary collaboration in environment and human health research (SEGH n.d.). Through its annual conferences and focused workshops (for example, the Multiple Links Towards Integrating Teams for Understanding of Disease and Environment (MULTITUDE) workshop (Ramsey and Stewart 2009)), in different regions internationally, professionals from disciplines across the earth/geochemical to medical/health sciences spectrum have been provided with the opportunity to meet and network with researchers from other disciplines.

Through these meetings, participants have been given opportunities to connect environmental and health disciplines, particularly human health. Specific feedback from this activity, as follow-up to one of these meetings, recorded, for example, the reflections of a hydrogeologist on his fresh realization of connections between physical and health sciences fields. Importantly, the need for collaboration between earth/environmental and health sciences professionals was appreciated and encouraged (Stewart et al. 2012). It also provided evidence of concern from the research community that such activity was problematic and not well received by peers.

Arsenic is a natural constituent of the environment. From 1995, it continues to rank as number one in the United States of America’s Agency for Toxic Substances Disease Registry (ATSDR)/Environmental Protection Agency (EPA) list of priority substances (Chou and De Rosa 2003; ATSDR 2015). In the environment, water is the component most vulnerable to toxic substances. This is because water bodies—rivers, streams, ponds, seas—are a sink for substances transported between environmental compartments, particularly the atmospheric and terrestrial systems. Water bodies (both surface and ground) serve as sources for drinking water, irrigation and recreation in many parts of the world (Kim et al. 2011). Drinking water is the highest single source of exposure to high arsenic levels by humans (ATSDR 2015; Yang et al. 2003; Chou and De Rosa 2003).

To investigate the positive feedback from participation in SEGH activities further (Stewart et al. 2012), we were encouraged to undertake an evaluation of the effectiveness of efforts to encourage multidisciplinary research beyond SEGH. Could truly multidisciplinary research activities in the fields relevant to SEGH be identified? How did the research methods adopted reflect the multidisciplinary team? Is a discipline “silent” in the methods developed and applied, or do specific approaches dominate the type of study design used? How can this knowledge further strengthen SEGH and the wider scientific community to promote multi- and interdisciplinary research?

In order to assess the nature of collaborative research efforts between earth and health scientists, we used a topical environmental issue of intense research effort as the focus namely arsenic in drinking water, with both wide spatial and temporal levels of investigation.

### Study designs in the earth and health sciences

**Fig. 1:**
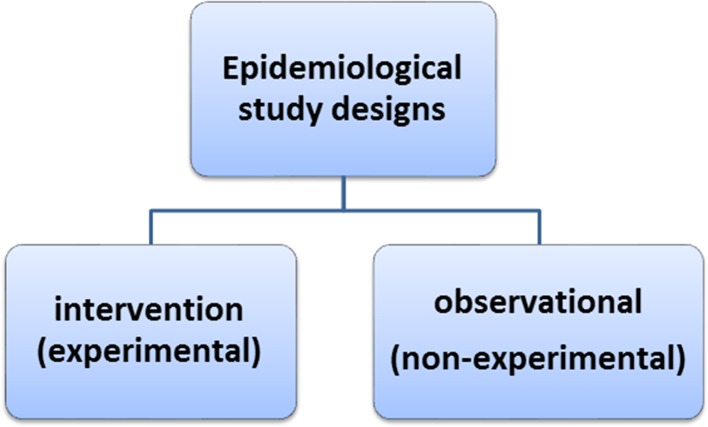
Classification of epidemiological (health) study designs

Earth and health scientists adopt different approaches when conducting research investigations. Earth scientists study the physical earth (atmosphere, water and land) by collecting data from field campaigns, designing computer simulations (models), running laboratory experiments to test response or reaction of inanimate materials, and undertaking appropriate replication to control for inherent variability (Earth Science Literary Principles n.d). Health scientists are concerned with developing an understanding of the multifactorial influences on human health, which include behavioural assessments as study endpoints. They adopt a range of methods in their investigation from quantitative and experimental through to observational and qualitative approaches. In moving from laboratory to field studies, they need to consider carefully their study methods in terms of how successful the data collection will be in providing robust population level evidence (i.e. epidemiology) and the impact the execution of the study may have on the nature of data collected (i.e. anthropological perspectives) (see Fig. 1) (Overview of Study Designs n.d). Laboratory analyses are an example of experimental methods. Non-experimental methods include data gathering through questionnaires, interviews, observation, focus groups.

## Methods and data sourcing

A desk-based analysis of published articles was undertaken. We wanted to highlight a period relevant to the work of SEGH and used as a starting point to the launch year for the Society’s journal Environmental Geochemistry and Health (1979). The analysis used readily available databases to provide a significant sample of the cross section of research outputs in the area, from which a more detailed analysis could be undertaken. A range of journals most frequently publishing on the topic was used to refine the sample to provide information on the relative number of articles published per year over the review period and to allow a comparison of the nature of publications in the first and most recent decades.

We subsequently classified articles sampled by author(s) affiliation(s) and later by authors’ field of research/discipline and to review study designs used in the articles co-authored by these individuals. Relevant articles were identified from a group of 20 journals reflecting the spread of journal publishers and output activity during a period 1979 (the launch of SEGH) to 2013.

The detailed methods were very similar to those used by Wang et al. (2014), Khan and Ho (2011), Hu et al. (2010) and Abejon and Garea (2015), where data (articles) were sourced using the online version of science citation index—expanded (SCI-Expanded) of the Web of Science from Thomson Reuters on 25 February, 2014. Open searching on “arsenic and drinking water” in the web of science for this period provided over 11,000 hits, so data reduction was required. Keyword searches were restricted to “drinking water”, “drinkable water”, “drinkable waters” and “drinking waterborne”, and “arsenic”, “arsenate” and “arsenite”, to compile a bibliography of relevant research articles. For each search “run”, we imputed a combination of one term from the group “‘drinking water’, ‘drinkable water’, ‘drinkable waters’ and ‘drinking waterborne’” and one term from the group “‘arsenic’, ‘arsenate’ and ‘arsenite’”. For example, “drinkable water and arsenic” or “drinkable waters and arsenate” or “drinking waterborne and arsenite”. Duplicate articles were identified and deleted. Details of articles downloaded were: title, names of authors, year of publication, author(s) affiliations/contact address, abstract, keywords and keywords plus. Not all articles obtained by imputing search terms provided above were used. Those used were selected using the following criteria:

1. articles reporting primary research only,
2. published in the “science and technology” field only and
3. published between 1979 and 2013.

Articles used were limited to research papers because it is expected they are products of original studies. Review papers, commentaries, editorials, and similar articles are believed to be summaries of original research, so were not considered further. Also, article searches were restricted to those in the science and technology field because the disciplines involved in this review are classed under the natural and medical/health sciences (OECD 2007). Articles from the social sciences, arts and humanities were not used. Furthermore, we only included articles from the 20 main science and technology journals publishing in the topic.

We acknowledge the limitation that our survey is based on data associated with the publications (author’s affiliations, addresses, etc.) and that fuller analysis based on author responses to direct questionnaires would provide a richer feedback. However, the scale of consultation and likely return rate might not produce any better detail than the approach used (Sivo et al. 2006). The data analysis produced over 800 articles from the subset of journals, which given the restriction on journal numbers to 20 compares favourably with the recent full bibliometric analysis on a research trend analysis of arsenic in drinking water subject by Abejon and Garea (2015) (1992–2012 ≥4000 articles).

### Classification of articles by authorship

Initially, our aim was to look at articles co-authored by environmental geochemistry and public health professionals alone. However, after a brief survey of articles, we realized that only a few articles were authored by these disciplines, either alone or in collaboration with other disciplines. Therefore, we broadened the coverage to the geosciences from environmental geochemistry and health sciences from public health. Then again, the coverage was extended to earth sciences instead of just geosciences. The term “earth science” was adopted because the classification guide used included geosciences and the guide did not explicitly explain which disciplines were covered by the term geosciences.

Articles were classified into one of nine categories based on details of author(s) affiliation(s) using the latest version of a standard reference document as a guide (the Revised Field of Science and Technology (FOS) Frascati manual DSTI/EAS/STP/NESTI (2006) 19/FINAL published by the Organization for Economic Co-operation and Development (OECD)). While there were other similar reference documents which could equally have been used (e.g. Australia and New Zealand Standard Research Classification (2008) and the Joint Academic Classification of Subjects (JACS) of the UK’S Higher Education Statistics Agency (HESA)), the OECD guide was chosen as the authoritative source due to publishing quality control by a recognized international organization with more than 200 countries as members.

Although the JACS document was more comprehensive than OECD’s, the OECD classification was chosen because of its international authorship. The task force which compiled the classification included members from the United Nations Education, Scientific and Cultural Organization (UNESCO), EUROSTAT (the statistical office of the European Union) as well as contributors from Australia, Norway, Portugal and Netherlands.

**Fig. 2:**
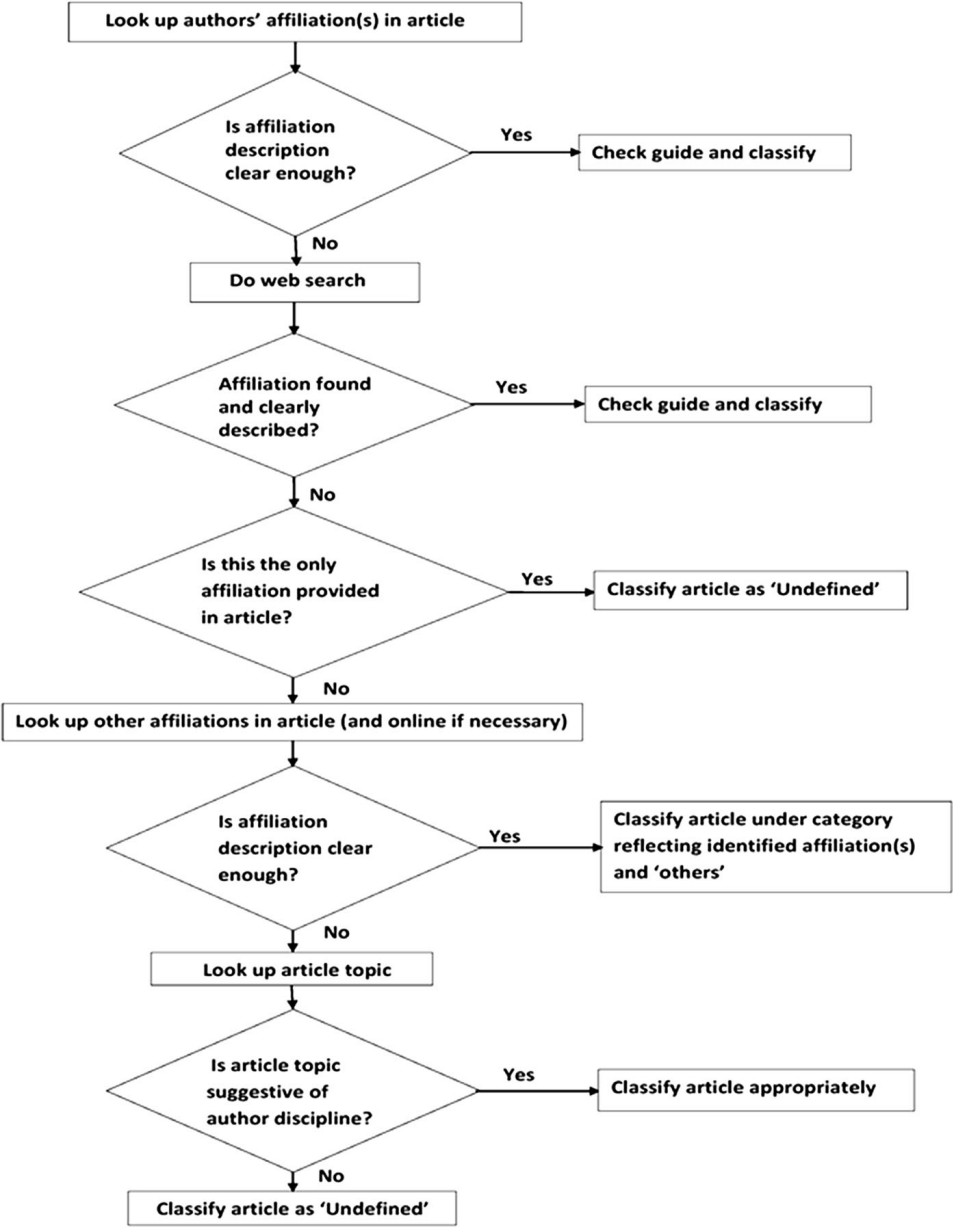
Decision flow chart used for article authorship classification

Initially, only the OECD guide was used to classify the authorship of an article. This guide was effective in classifying an article only when author(s) affiliation(s) were clearly described and easily interpreted. For articles that had ambiguous affiliations and could not be easily interpreted by the guide, other means like web searches (used by Stewart et al. 2012) were sought to establish the author(s) discipline. Later, due to our inability to maintain a reproducible order of classifying the articles and to ensure maximum objectivity, it was necessary to develop an algorithm for the classification process (Fig. 2). After construction of the algorithm, the whole review process was restarted. Apart from establishing order, the algorithm solved a number of problems encountered during the classification process. Some of the problems included difficulty with classifying articles:

i. With affiliations not clearly stated in the OECD guide,
ii. Where a web search was not sufficient to establish the author’s discipline,
iii. Or where clear affiliation information was provided for only some authors in an article.

The algorithm specifies three decision points to be used to classify an article’s authorship:

i. Affiliation as described in the article and interpreted by the OECD guide,
ii. Affiliation as described after an online search and interpretation by the OECD guide
iii. Article topic

The third decision point (article topic) was seldom used, because we found that article topics do not always reflect author(s) discipline(s).

However, even with the use of the decision algorithm and OECD guide, some articles could not be classified appropriately. This was due to reasons such as:

i. Insufficient information about author affiliation and sometimes author institution in article,
ii. Some affiliations were described in languages other than English and could not be clearly interpreted using the Google Translate^®^ online software,
iii. Non-functioning and poorly managed institution web addresses,
iv. Research students without established disciplines or research interests/fields,
v. Authors who have changed institutions and those who were temporary staff of institutions where they were when article(s) were published,
vi. Retired or deceased authors,
vii. Ambiguous affiliations and disciplines, for example, “medical geology”; “chemical sciences in the faculty of health”,
viii. Broad fields of study, such as environmental science, which can include social, earth, and health aspects.
ix. Government agencies and research institutes involved in a wide variety of research and with staff from widely different fields, for example, United States Environmental Protection Agency (USEPA) and Biomed Incorporated USA.

The whole classification process was completed twice. The first round focused on using primarily the author(s) affiliation/contact address as provided in the article and online. Authorship of articles was classified under one of nine categories;

i. Earth sciences only
ii. Earth sciences and others
iii. Health sciences only
iv. Health sciences and others
v. Earth and health sciences only
vi. Earth, health sciences and others
vii. Others
viii. Undefined
ix. Not full article and duplicates

**Note:** “Health sciences” in this study covers medical/health sciences. The “earth sciences only” category refers to articles authored by scientists affiliated only to earth sciences disciplines or research fields. Similarly, the “health sciences only” category refers to articles authored only by health scientists. The “earth sciences and others” and “health sciences and others” categories cover articles co-authored by earth scientists alongside authors from disciplines outside the earth and health sciences, e.g. engineering, chemistry, economics and articles co-authored by health scientists and authors outside the health and earth sciences, respectively. The “others” category contains articles authored by disciplines outside both the earth and health sciences. Affiliations that were not found in the classification guide and could not be identified through online search were classed as “undefined”. “Not full article and duplicates” category contains articles that are not full research articles such as commentaries, corrections and duplicates that were omitted by the bibliography software (Endnote^®^) during the duplicate checking and deleting process.

### Classification of articles by authors’ disciplines/research interests

The second round of classification focused only on those articles classified as “earth and health sciences only” and “earth, health sciences and others” categories, since these were the main categories of interest for the review. Here, the classification criterion was extended to include authors’ specific research fields in order to identify articles with at least one earth scientist and health scientist as author. Originally, we did not intend to include authors’ specific research fields as a classification criterion, but after discovering that an authors’ affiliation does not always reflect their discipline, we refined the classification criteria. After the first classification round (affiliation-based), we searched online to establish, where possible, the actual discipline/research fields of authors. During this search, we used:

i. The authors’ research interest or areas of research and
ii. The authors’ field of doctorate study (for authors’ with PhDs)

The online search was completed through:

i. Websites of authors’ affiliations
ii. LinkedIn^®^ and Research Gate^®^
iii. Other online professional networks found for the relevant author

LinkedIn^®^ is the world’s biggest professional network (LinkedIn n.d). Research Gate^®^ is an online community where scientists meet to ask and answer questions, share research articles and connect with collaborators (Lin 2012).

### Classification of study designs

Study designs adopted in the articles which highlighted collaboration between earth and health scientists were classified using guides from WHO (2001) and Earth Science Literary Principles (n.d.). We were able to undertake this classification using information provided in the methods section as well as from the aim(s) of the research (provided in the introduction section) of the articles.

## Results

### Top 20 journals publishing in arsenic in drinking water research from 1979 to 2013

**Fig. 3:**
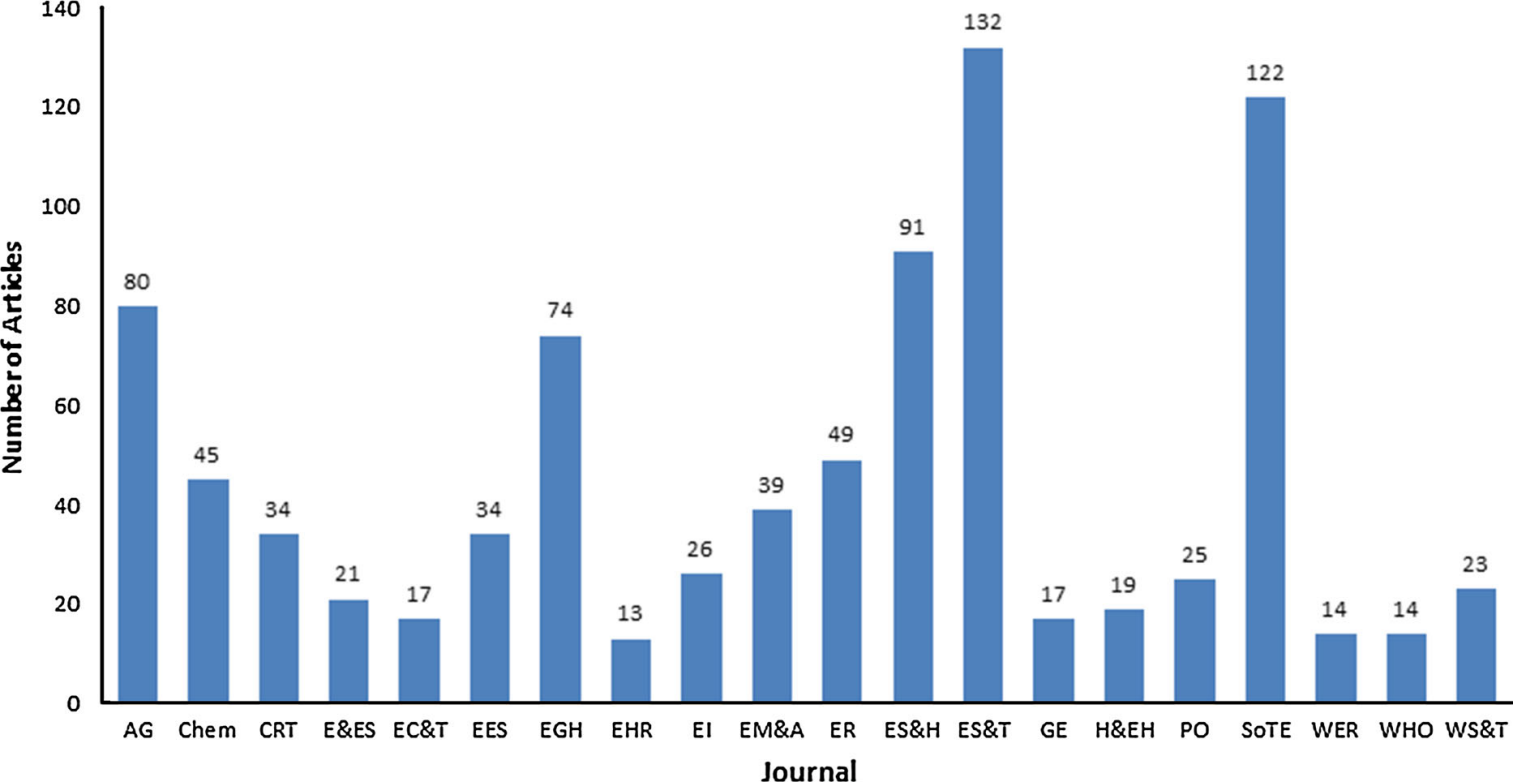
Top 20 journals publishing in arsenic in drinking water from 1979 to 2013, based on criteria described in the text

A total of 889 articles were identified over the 35-year period. These articles were published in 20 journals covering a wide range of relevant subjects. Environmental Science and Technology (ES&T) had the highest number of articles (132 (15%)), while International Journal of Environmental Health Research (EHR) had the lowest number with only 13 (1%) (Fig. 3). As part of a pilot study, the aims and scope of the twenty journals identified had been searched. Combining that study with this, we noted that the top three journals that published the highest number of articles are very multidisciplinary in scope, covering virtually every area of environmental research. They are Environmental Science and Technology (ES&T), Science of the Total Environment (SoTE) and Environmental Science and Health (ES&H). Other journals were more restricted in scope—covering basically earth and medical/health sciences, for example Applied Geochemistry (AG) and Environmental Geochemistry and Health (EGH) (Full names of other journals are available in Table 1). The nature of study types published by individual journals (Table 2) reflects editorial focus.

**Table 1:** Journal abbreviations and full titles

Abbreviation	Full journal title
AG	Applied Geochemistry
Chem	Chemosphere
CRT	Chemical Research in Toxicology
E&ES	Ecotoxicology and Environmental Safety
EC&T	Bulletin of Environmental Contamination and Toxicology
EES	Environmental Earth Sciences
EGH	Environmental Geochemistry and Health
EHR	International Journal of Environment Health Research
EI	Environment International
EM&A	Environmental Monitoring and Assessment
ER	Environmental Research
ES&H	Journal of Environmental Science and Health Part a—Toxic/Hazardous Substances & Environmental Engineering
ES&T	Environmental Science and Technology
GE	Journal of Geochemical Exploration
H&EH	International Journal of Hygiene and Environmental Health
PO	Plos One
SoTE	Science of the Total Environment
WER	Water Environment Research
WHO	Bulletin of World Health Organization
WS&T	Water Science and Technology

**Table 2:** Summary of study/research designs adopted in articles co-authored by earth and health scientists and other disciplines in arsenic in drinking water research (articles selected from those published between 1979 and 2013)

Authors	Summary of research activities	Study designs
Peters et al. (1999)	Sampling water and rocks, and analysis of As content	Non-experimental–descriptive (field)
Kim et al. (2000)	Sampling of core soil samples and determination of total arsenic content using graphite furnace atomic absorption spectrophotometer (GF-AAS) evaluating effects of ions on leaching of arsenic investigation of the role of bicarbonate ion in arsenic dissolution	Non-experimental–descriptive (field) and experimental
	Exploration of effect of aerobic and anaerobic conditions on the rate of arsenic leaching	
	Elucidation on the effect of hydrogen ion concentration on arsenic leaching	
	Investigation of arsenic release from sandstone samples	
	Investigation of the influence of sodium bicarbonate and sandstone samples on the stability of arsenic species	
	Assessment of arseno-carbonate complex using ion chromatography	
Matschullat et al. (2000)	Water, urine, soil, sediment and mine tailing samples were collected	Non-experimental–analytical (field)
	Questionnaire was used to collect information on age, gender, place of birth, period of residence in sampling site, nutrition habits and health status of subjects	
	Arsenic, mercury and cadmium contents of samples were determined using atomic spectroscopy (e.g. flame AAS, HG-AAS and GF-AAS)	
Van Geen et al. (2002)	Collection of water samples from 4997 tube wells, data gathered on number of tube wells, composition of household residents, date of installation and the depth of the wells using questionnaire, determination of well location using hand-held GPS and determination of total As concentrations using GF-AAS and high-resolution ICP—MS	
	Non-experimental–descriptive (Field)	
Reimann et al. (2003)	Collection of water samples from deep and shallow wells, springs, hot springs and rivers and determination of concentrations of 65 chemical elements (including F) using ion chromatography (for anion analysis), ICP–OES and ICP–MS to obtain quantitative data on elemental concentrations	Non-experimental–descriptive (field)
Hira-Smith et al. (2003)	Provision of technical assistance in terms of the engineering design and construction of hand dug wells	Classification not available
Van Geen et al. (2005)	Collection of groundwater samples from a total of 6874 wells from year 2000 to 2003 determination of arsenic concentration using field kits and laboratory instruments	Non-experimental–descriptive (field)
Ayotte et al. (2006)	Modelling of the likelihood that arsenic levels in bedrock wells are ≥5 µg/L using logistic regression	Modelling study
Dodd et al. (2006)	Determination of rate constants for the reactions of As (III) with oxidants such as free available chlorine (FAC), chloramine (NH_2_Cl), dichloramine (NHCl_2_) and ozone (O_3_)	Experimental
	Calculation of stoichiometry of the reactions between As (III) and each oxidant	
Hira-smith et al. (2007)	Measurement of total and faecal coliform counts in water samples collected from monitored dugwells using membrane filtration method determination of concentrations of 13 metals (including As) in the dugwells using flow injection atomic absorption spectrometry (AAS)	Non-experimental–descriptive (field)
Jakariya et al. (2007)	Collection of water samples from particular tube wells in the study area analysis of As contents using the field kits and in the laboratory using HG–AAS determination of geographical coordinates of sampled wells using GPS receivers	Non-experimental–descriptive
Katsoyiannis et al. (2007)	Sampling of groundwater	Non-experimental–descriptive (field)
	Determination of sulphate, chloride and nitrate, (NO_3_–N) using ion chromatography	
	Determination of total arsenic using hydride generation atomic fluorescence spectrometer (HG-AFS)	
	Determination of uranium, selenium and antimony using ICP-OES	
	Determination of alkalinity and total hardness by titration	
	Determination of total organic carbon (TOC), total nitrogen (Total-N) with a TOC analyser	
	Arsenic speciation studies	
Kocar et al. (2008)	Digging of sample wells, collection of water samples from the sample wells and determination of As, alkalinity, and organic carbon contents of the water samples	Non-experimental–descriptive (field)
McKnight-Whitford et al. (2010)	Collection of groundwater samples and analysis of As contents using high-performance liquid chromatography–inductively coupled plasma mass spectrometry (HPLC–ICPMS)	Non-experimental–descriptive (field)
Pearce et al. (2010)	Data gathering on children’s diet and leisure activity using questionnaire survey, collection of soil samples from study sites and toenail samples from the subjects (children), determination of As concentrations in soil and toenail samples, As speciation studies and statistical analysis of data	Non-experimental–descriptive (field)
Nagar et al. (2010)	Collection and characterization of Fe- and Al-based water treatment residual (WTR) samples	Experimental
	Sample characterization for organic matter content, electrical conductivity, solution pH, etc.	
	As (V) sorption experiments in the absence and presence of competing ligands and complexing metal	
	Surface complexation modelling was done using constant capacitance model (CCM) to position As (V) sorption for both Al- and Fe-WTR surfaces in the single ion (As or P) and binary (As + P) systems, and statistical analysis of data	
Fillol et al. (2010)	Urine and soil samples were collected	Non-experimental–analytical and descriptive
	Questionnaires were used to collect data from subjects in sampling area	
	Creatinine concentrations in urine samples were determined	
	Chemical species of As and speciation studies in soil samples were undertaken	
	As content in urine, in soil and in atmospheric particulate matter were determined	
	Data on As concentration of water samples in the area were obtained from results from routine controls by the Direction Départementale des Affaires Santaires et Sociales (DDASS), an administrative body engaged in public health policy, immigration, disability and protection of the vulnerable in France (Santémédecine.net n.d.)	
	Statistical analyses of results	
Wu et al. (2011)	Data collection on incidence of childhood diarrhoeal disease from records of an extensive Health and Demographic Surveillance System (HDSS) programme covering the study area, sampling of water from 10, 869 wells in the area, and determination of As contents using HG–AAS including field kits	Non-experimental–analytical (field)
Escamilla et al. (2011)	Use of secondary data from the HDSS programme survey of all tube wells, latrines and household locations	Non-experimental–analytical (field)
Van Geen et al. (2011)	Sampling of water from 125 wells determination of As contents measurement of precipitation and waters levels to indicate the rate of surface and groundwater recharge	Non-experimental–descriptive (field)
Bhattacharya et al. (2011)	Water sampling from 61 tube wells out of the 13,269 functional ones in the area, determination of As (total) concentrations in the sampled waters and analysis of As concentration data to comprehend the temporal and seasonal irregularities in dissimilar concentration ranges	Non-experimental–descriptive (field)
Maity et al. (2012)	Collection of 52 groundwater samples representing about 10% of the available tube wells in the areas using acid-washed 500-mL polyethylene bottles, collection of hair, toenails and urine samples from subjects determination of aggregate As content in groundwater, hair, nails and urine using a Fluorescence Atomic Analyser	Non-experimental—analytical (field)
Kozul-Horvath et al. (2012)	Adjusting mated mice to a rodent diet labelled AIN–76A, grouping of mated mice into control and exposure groups and exposure to different As doses (male mice were not exposed to As before mating), further grouping after birth of female mice in the control and exposure groups and exposure to different As doses total As concentrations were determined by ICP–MS	Experimental
Halder et al. (2012)	Collection of water, rice, vegetables and urine samples and determination of their As concentrations	Experimental
George et al. (2012)	Collection of water samples and determination of their As concentrations	Non-experimental–descriptive
Rango et al. (2012)	Sampling of groundwater, questionnaire survey and examination and of DF cases and determination of F, As, bicarbonate (HCO_3_^−^), etc., contents of sampled water	Non-experimental–descriptive (field)
Asante et al. (2012)	Collection of water from boreholes, wells, spring, stream and tap and human urine samples Use of questionnaire to collect biodata from selected subjects	Non-experimental–descriptive (field)
	Determination of pH and conductivity in water samples	
	Determination of concentration of various metals in water and urine samples using analytical instrumentation such as ICP–MS, AAS	
	Statistical analysis of results	
Halder et al. (2013)	Data gathering using questionnaire-based survey analysis of rice samples eaten by residents of rural areas of West Bengal, India	Non-experimental–descriptive

### Number of articles published per year

**Fig. 4:**
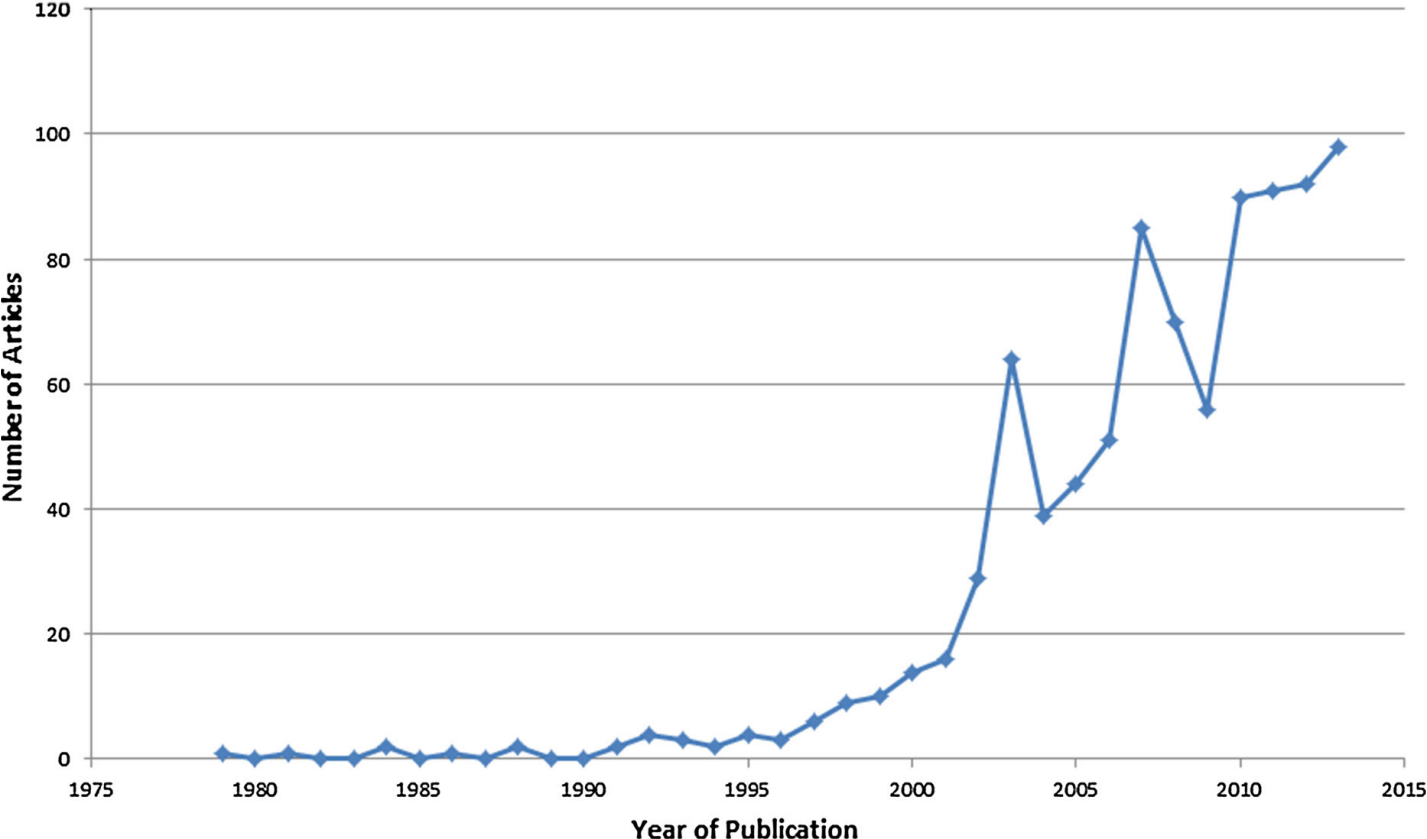
Set of arsenic in drinking water research articles published from 1979 to 2013

Figure 4 presents the number of articles published per year. In 1979, the first year of review, only one article was published. The next year, no article was published. Again, only one was published in 1981. The largest increase in the number of published articles occurred between 2002 and 2003: 29 to 64 articles, an increase of more than 220%. The highest number of articles published in a single year was 98 in 2013, the last year in the review period.

### Comparison of nature of publications in the first and last 10 years (1979–1988 versus 2004–2013)

**Fig. 5:**
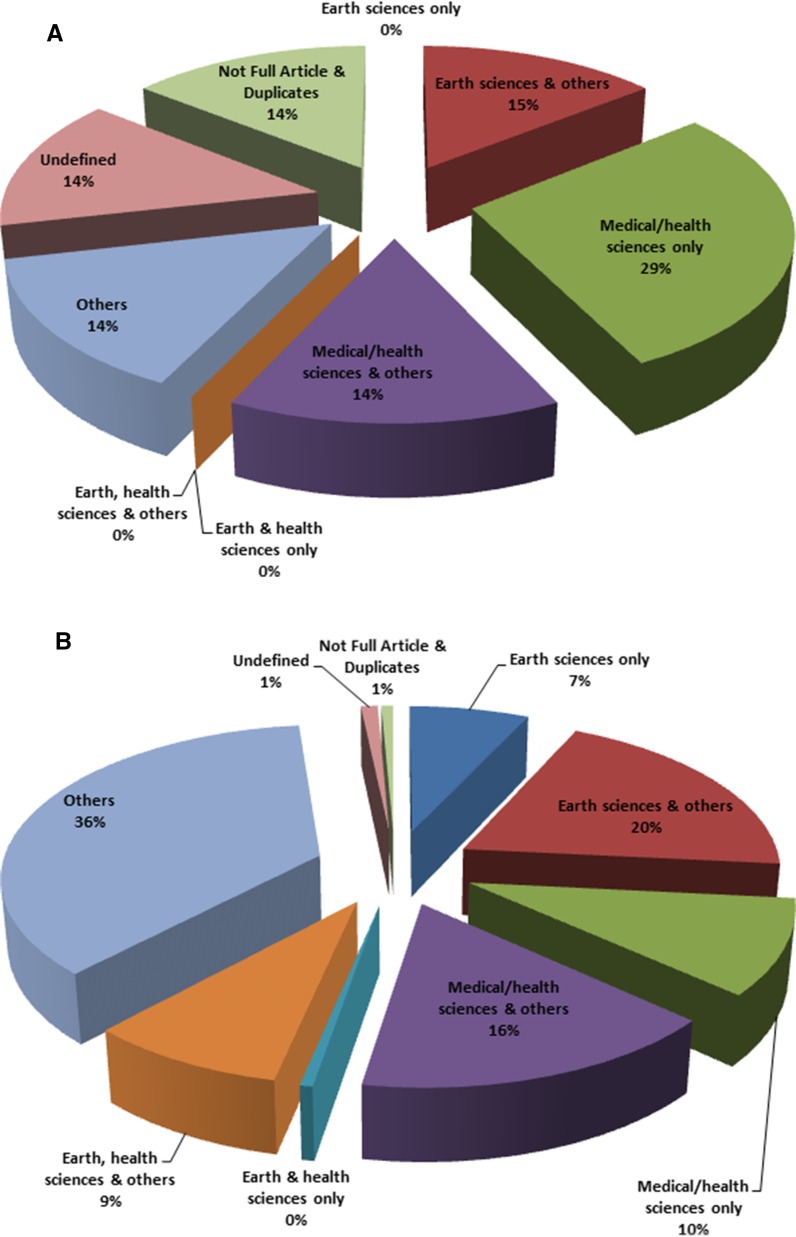

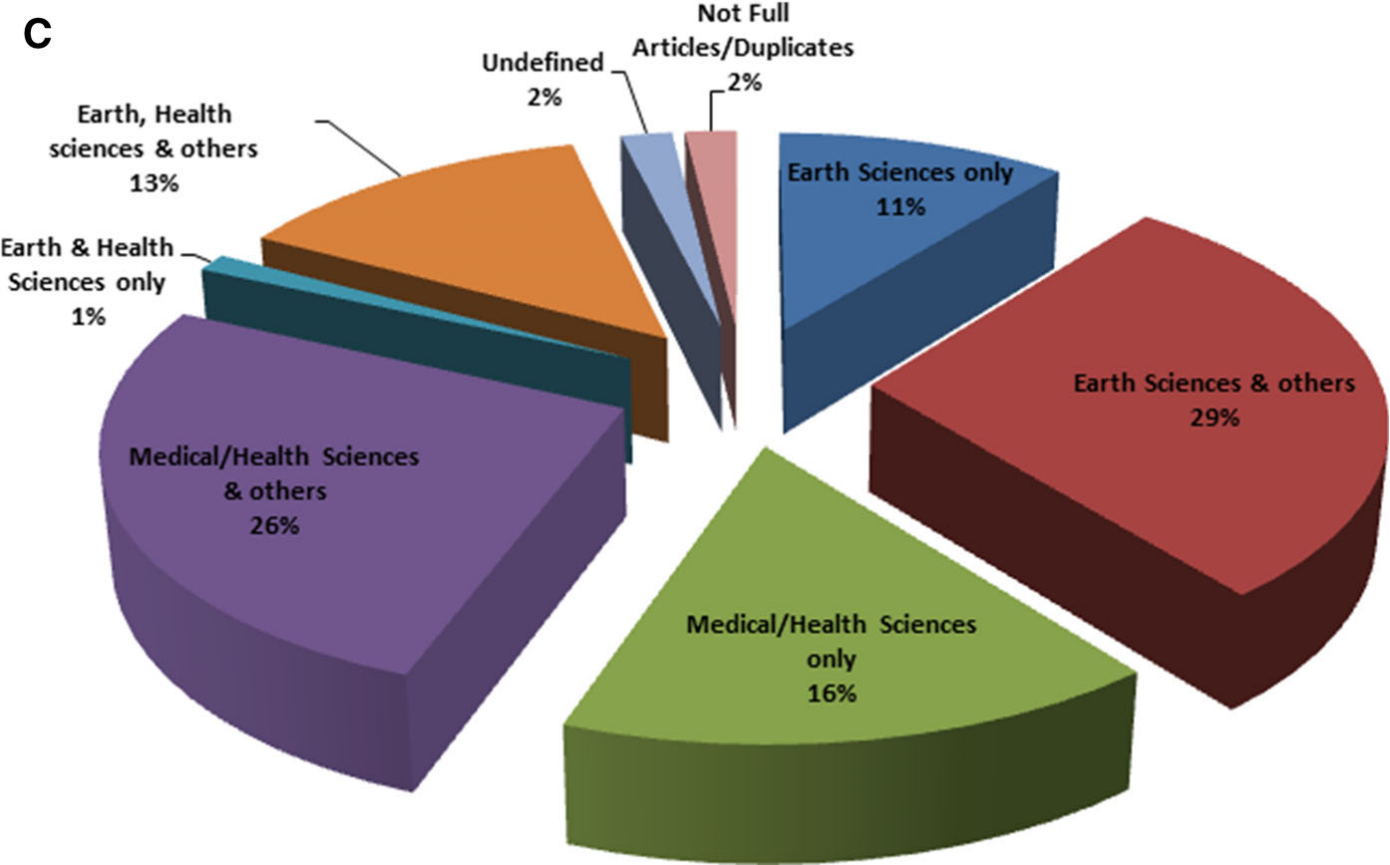
Classification of articles in arsenic in drinking water research by author affiliation. **a** First 10 years—1979 to 1988, **b** last 10 years—2004 to 2013, and **c** over the whole review period—1979 to 2013 (**c**)

Changes in the nature of publications over the review period are shown in Fig. 5a, b. The pie charts provide a graphic comparison of the nature of publications between the first and last 10 years of review.

### Distribution of article authorship

After classifying the articles by authors’ affiliation only, the “earth and health sciences only” and “earth, health sciences and others” categories had 7 (<1%) and 77 (8.7%) articles, respectively. Together, both categories had 84 (~9%) out of the 889 articles classified (14% when the “others” category is excluded) (Fig. 5c). Classifying the articles in these categories by the disciplines of the authors, only 28 (33%) of the 84 articles had at least one earth and at least one health scientist on the team of authors. The remaining 56 articles were classed as “others”. This “others” class represents articles not having at least one earth and not at least one health scientist jointly on the authors’ list. Some had earth scientists with other authors outside the earth and health sciences, other such articles had health scientists and other authors outside the earth and health sciences, yet others had either earth or health scientists working with professional from other fields. Of these 56 articles, nine articles could not be classified because there was insufficient information about the authors. Further, Fig. (28) represents only 3% of the total number of 889 articles. This indicates that level of collaborative research between earth and health scientists’ experts using arsenic in drinking water studies as a case study is low. We note that the database software used was not 100% effective in filtering out unwanted article types (Fig. 5c).

### Classification of study designs used

**Fig. 6:**
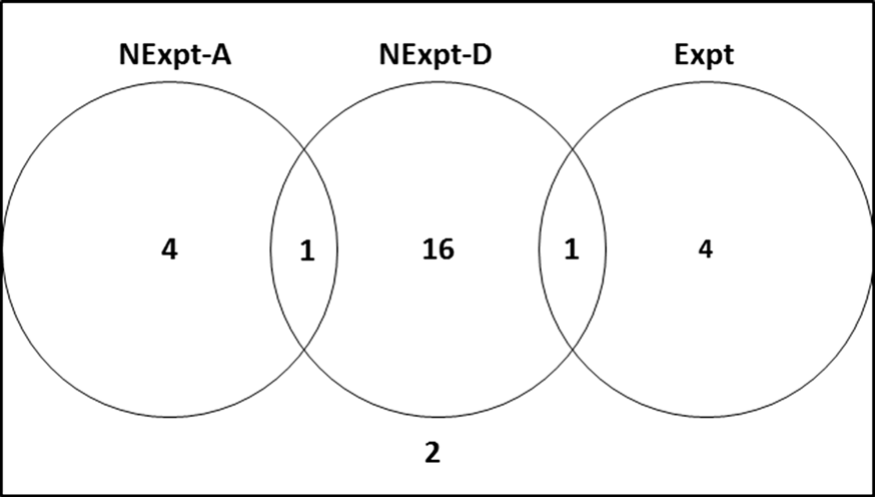
Venn diagram showing distribution of study designs in 28 articles co-authored by earth and health scientists in arsenic in drinking water research published from 1979 to 2013 (*NExpt*-*A* Non-Experimental Analytical*, NExpt*-*D* Non-experimental Descriptive*, Expt* Experimental)

Of the 28 articles of interest, 16 articles involved descriptive type of non-experimental study design, four adopted an experimental type of study design, one combined descriptive non-experimental with experimental type of research design, one combined descriptive and analytical non-experimental research designs, one involved modelling, while the study design used in one of the articles could not be classified as either experimental or non-experimental research (Fig. 6).

## Discussion

Our selection of articles from the top 20 journals by number of papers is a reasonable pool of data to investigate relationships and identify any trends in collaborations.

### Top 20 journals publishing in arsenic in drinking water research from 1979 to 2013

The study by Abejon and Garea (2015) used less restricted search terms although over a more restricted time period resulting in 4143 hits. We restricted our subset to original research articles to allow us to investigate research teams engaged in data generation. In a review article on the toxicological effects of arsenic, 1809 articles were identified (Khan and Ho 2011). Our more restricted view was necessary to allow us to collect a profile “snap shot” of research team assessments rather than productivity.

The most important source of arsenic poisoning over the past decade or so has been the water contamination in Bangladesh and South-East Asia. Also, the UK interest in soil arsenic contamination is a risk-based approach, with no demonstrated direct effect on health at this point. Concentrating solely on the Asian or the UK situation would give very different numbers of papers, with a possible different mix of disciplines involved.

### Rate of change in number of articles published per year

The number of articles published per year varied, from one in 1979, none in 1980, and one in 1981 to 98 in 2013—the highest number of articles in a single year. The relative growth in interest signified by changes in publication number is comparable to the broader assessment of Khan and Ho (2011), albeit absolute numbers are very different as explained above.

### Comparison of the nature of publications

More articles were published in the last 10 years than in the first ten. One reasonable explanation for this observation is that cases of arsenic poisoning became more prevalent in the twenty-first century than before. The first arsenic disease patients from Bangladesh were identified in West Bengal, India, in 1987. Later in 1993, it was confirmed that water in tube wells in the Chapai Nawabganj district, north-western part of Bangladesh, were contaminated with arsenic (Smith et al. 2000). These and subsequent discoveries undoubtedly increased research interest in the topic. In the first 10 years, almost all categories except medical/health sciences had the same number of articles—one article (representing 14%) each (Fig. 5a). This perhaps suggests that arsenic enjoyed equal attention from these fields then. In the last decade of review, the “others” category had the highest number of articles (260 out of 838). This probably reflects a greater interest in arsenic arising in fields outside the earth and health sciences in later years.

### Distribution of article authorship

These classifications were based on the assumption that all authors listed in each article actually contributed to the studies. Authorship rules are often difficult to assess, given the nature of funding scientific studies and collaborations based on organizational arrangements. Consequently, a possible confounder in this approach is that some cited authors may not have made significant contributions to the delivery of the study reported. An important inference from the result is that affiliations do not necessary reflect an author’s discipline or research interest. This result is similar to the finding by Stewart et al. (2012) that only about nine (28%) of the 32 articles reviewed in a study appraising the impacts of multidisciplinary meetings organized by SEGH had both health and environmental sciences professionals as co-authors. Though environmental science as a field of study covers other disciplines including some earth sciences disciplines and it is not clear which research topics were considered in Stewart et al. (2012), results from our study and from Stewart et al. (2012) indicate that level of collaborative research between earth and health sciences experts based on co-authored papers may be very low.

### Classification of study designs used

Nevertheless, there are clear cases where multidisciplinary teams were developed for environmental and health-related research, composed of professionals both from the earth and health fields as well as others from outside these fields. We give four examples: a report on multidisciplinary actions taken in Uruguay to reduce human exposure to lead (Pb) was based on work by a team that is comprised of chemists, social workers, delegates from communities, the energy and mineralogy ministries and health scientists (paediatricians, toxicologists) amongst others (Cousillas et al. 2012).

Similarly, Ahsan et al. (2006) reported that a prospective cohort study of a Bangladeshi population exposed to a wide dose range of arsenic was carried out by a multidisciplinary team including social scientists, earth scientists and physicians. Further, a study aimed at integrating medical and geochemical methods to understand the probable impact of waste disposal on human health in Lovke, Croatia, was undertaken by a team composed of professionals from the health sciences (staff from a general hospital and a general medical service) and earth sciences (Franciškovic–Bilinski et al. 2007).

Finally, the Superfund Basic Research Program (SBRP) of the National Institute of Environmental Health Sciences (NIEHS) in the USA is involved in studies to tackle arsenic contamination in drinking water. Since 1990, the SBPR has been bringing together researchers from the fields of ecology, engineering, mathematics as well as the health sciences to investigate the fate, transport and remediation methods for arsenic in groundwater and soils (Suk and Holden 2004). Some of these studies appear not to feature earth or health personnel. A study aimed at proving the applicability of two contrasting biological assays for investigating coastal areas susceptible to human activities had researchers from institutes of agricultural biology and biotechnology, chemistry and biophysics (Frassinetti et al. 2012).

Research study designs used in a multidisciplinary programme may reflect research design traditions from only one of the disciplines involved, or, the study design adopted may be hybrid but not traceable to a particular discipline. Yet in others, research designs from the different disciplines involved in the studies may be more equitably reflected.

In a multidisciplinary study aimed at integrating geochemical and medical methods to enquire into the probable impact of waste disposal on human health, Frančiškovič-Bilinski et al. (2007) reported a study design that centred on sampling and chemical analysis (instrumental) of solid wastes and stream sediments. This study design, classified as a typical field study approach, is common in the environmental sciences, i.e. by environmental chemists, geologists, geochemists (McLelland n.d.). No mention was made in the study of the methods used to investigate the connection between the waste disposed and observed or suspected health impacts. The only comment on any health-related study in this research article was made in the results section. The statement revealed that the health study was undertaken in a preliminary study. It reads “A preliminary study was performed to make an overview of the health situation of inhabitants of Lokve, who have been exposed to barium for a long time”.

A report by Cousillas et al. (2012) highlights a multidisciplinary project on lead pollution in Uruguay which involved a laboratory of environmental hygiene and the Ministerial Division of the Environment in the assessment of lead, chromium and cadmium in contaminated soils in parts of the country and included the Ministry of Health collecting soil as well as blood samples from slums for analysis. However, the article was not explicit about the exact disciplines of those that carried out the soil sampling.

It is critical that multidisciplinary articles describe clearly appropriate methods reflecting the disciplines of all contributing authors. Reviewers need to consider this in assessing the quality of submissions for publications and where research methods may have been “borrowed” from one discipline and applied by others. Clear scrutiny of the study design and interpretation needs to be emphasized. In many cases, even routine scientific methods are frequently poorly described.

In contrast, Ahsan et al. (2006) reported a study design which reflected contributions from the research methods common to the different disciplines in the research team (as determined by their affiliations). The study aimed to undertake background work in preparation for a cohort study of a population exposed to As in Bangladesh. The study design was a mix of different research approaches. It included sampling and analysis of water from tube wells (possibly by earth scientists in the team). According to the report, the chemical analysis was carried out in the Geochemistry Research Laboratory of Columbia University, USA by geochemists or other earth scientists. Though the report did not identify whether sample collection and subsequent analysis of venous blood and urine were performed by medical/health team members, it is believed that currently accepted practice in publically funded research would require ethical approval for this type of work and appropriately skilled/experienced personnel for blood and urine sampling.

Physical sampling and chemical analysis were not the only activities performed in the study, cohort members were also recruited. A cohort refers to a group of people with similar characteristics selected for the purpose of investigating the health effects of exposure to a particular substance. Such cohort studies are an example of observational methods used in epidemiological research (WHO 2001; Overview of Study Designs n.d.), which are common in work undertaken by health scientists and fairly limited in the earth sciences. The report notes that each recruitment and data collection team for the proposed cohort study had a field physician along with another unidentified team member in a two-member team.

Equally, in a multidisciplinary approach to prove the applicability of two different biological assays for observing a coastal area susceptible to anthropogenic inputs, Franssineti et al. (2012) adopted a study design that combined chemical and biological analyses. These analyses types were clearly reported under methods. It is assumed that the chemical analyses were performed by the chemists in the team, while the bioassays may have been undertaken by the biotechnologists in the team. However, as was highlighted in the methods of Franciškovic–Bilinski et al. (2007), instrumental analysis of metal content of the sediments by electrothermal atomic absorption spectrometry (ET-AAS) may have been undertaken by suitably trained personnel irrespective of scientifically defined disciplines.

It is clear that in multidisciplinary research projects in the earth and health sciences, study designs are adopted that reflect either input by only one discipline or integrate traditional research designs of the different disciplines on the research team. It also raises the question of the merits of study design and consequently value of data produced, where it is not clear that the protocols used were developed by suitably informed, experienced and engaged team members (Dickinson et al. 2009).

A descriptive non-experimental type of study design was the one most adopted—16 out of 28 articles (57%). Descriptive non-experimental research involves answering the question of “what is” rather than checking the correctness of a hypothesis (or finding out “how”) (WHO 2001). As regards arsenic, such studies mainly concerned determination of arsenic and other metal ion concentration in samples. It was clear that activities such as sampling, and sample treatment for instrumental analyses dominated the environmental study design sections.

Examples of samples collected include water, soil and urine. Instrumental analyses undertaken included atomic absorption spectrophotometry (AAS), graphite furnace atomic absorption spectrophotometry (GF-AAS), hydride generation atomic absorption spectrophotometry (HG-AAS), inductively coupled plasma optical emission spectrometry (ICP-OES), inductively coupled plasma mass spectrometry (ICPMS) analyses. This variety is a possible explanation for the inclusion of scientists from different fields in the research team. As noted elsewhere in this paper, it would be good to have similar levels of detail about the relevant health aspects of the studies.

## Conclusion and recommendations

Findings from this study show that the level of collaborative research, in terms of co-authored research papers published in the top 20 journals in the arsenic in drinking water topic from 1979 to 2013 by earth and health scientists, is low. Even with the recognized need for collaborative research between these disciplines and with others, as the case may be, very little “multidisciplinarity” is probably achieved in practice. In articles with evidence of multidisciplinary research, research designs adopted were such that did not require discipline-specific skills. Collaboration in research requiring discipline-specific skills in multiple disciplines is uncommon.

Studies on the determination of As contents in water, soil, air, to mention a few, are very important as they can help in better understanding exposure levels and in guiding decisions on remediation. While some of the studies reviewed measured exposure levels, only one study investigated a remediation method (Nagar et al. 2010). However, this was a laboratory-based experimental research, not a field remediation study. In situ remediation methods are needed, because of the predominance of natural source of As release (Vall et al. 2012; De Rosa 2003). It is not enough that exposure levels are measured or that the sources of As release is known; it is essential that appropriate clean-up methods (to acceptable limits) are researched. Affected communities will be less interested in, for instance, how a toxic substance is released into their water and how much of it they are exposed to. They will be more concerned about cleaning up their water.

Earth and health scientists, alongside professionals from other disciplines, can apply previous knowledge in risk assessment for instance, in developing suitable remediation methods. As multidisciplinary teams find ways to make decisions, it is imperative that members of the team understand that they are laymen as far as knowledge, skill and expertise in disciplines outside theirs is concerned (Mahoney et al. 2015). This situation can cause communication problems which can develop into misunderstanding (Stewart et al. 2012). Fortunately, Mahoney et al. (2015) provide some proven suggestions as to how this problem can be solved. First, they recommend that parties (multidisciplinary team members) involved should endeavour to listen to each other. Second, they suggested that communication should be transparent, honest and effective. For investigations that require interaction with the public, Mahoney et al. (2015) suggest that these same recommendations can ensure success, if applied.

Similarly, although we did not assess the statistical aspects of the papers we reviewed, statistical support from knowledgeable specialists should be sought. Complex statistics used without expert advice may be used wrongly. This is a long-recognized interdisciplinary issue in the medical literature (Altman et al. 2002).

Results from our study have implications for universities, scientific communities, research institutes and government agencies and other agencies involved in research, control and remediation of contaminated situations, whether from arsenic or from other pollutants. There is no reason to believe that the issues we have highlighted in multidisciplinary work around arsenic are unique to that element.

Early career researchers should be encouraged to interact with students and professionals from disciplines outside their own from an early stage in their career. Evidence is emerging that at undergraduate level skills required in modern work environments need to be multidisciplinary and that as information and communication technologies develop, access to information across a variety of domains creates opportunity to solve complex problems, by combining approaches form many fields. However, the skills required in translating, interpreting and understanding the information to inform decision making need the right educational context (Jacob 2015).

Scientific communities, especially those taking a multidisciplinary approach to environmental issues, need to understand that they may have to do more to encourage collaboration across disciplines, while research institutes and government agencies also need to increase efforts to encourage boundary-crossing between disciplines, new learning experiences and acknowledge the strong value to wider societal problems. Because the disciplinary language barrier may inhibit multidisciplinary collaboration (Huby and Adams 2009), developing multidisciplinary collaboration at the design stage of the research is essential to smooth the rough edges of language barriers and contrasting, traditional research designs. Research into the effect of the environment on health (e.g. non-particulate air pollution, chemical mixtures, contribution to chronic diseases such as diabetes and dementia) needs greater multidisciplinary approaches.

Finally, we call upon authors, journal editors and reviewers to ensure that papers that cross-disciplinary divides include clear descriptions of any and all methods used. These descriptions should be given in a way appropriate to the discipline of the work undertaken. For example, in the methods section, geochemical studies should be described in the usual geochemical manner, while the description of health studies should be of a standard acceptable in health journals.
